# Dose-response in modulating brain function with transcranial direct current stimulation: From local to network levels

**DOI:** 10.1371/journal.pcbi.1011572

**Published:** 2023-10-26

**Authors:** Ghazaleh Soleimani, Rayus Kupliki, Martin Paulus, Hamed Ekhtiari

**Affiliations:** 1 Department of Psychiatry, University of Minnesota, Minneapolis, Minnesota, United States of America; 2 Laureate Institute for Brain Research, Tulsa, Oklahoma, United States of America; University of Nottingham, UNITED KINGDOM

## Abstract

Understanding the dose-response relationship is crucial in studying the effects of brain stimulation techniques, such as transcranial direct current stimulation (tDCS). The dose-response relationship refers to the relationship between the received stimulation dose and the resulting response, which can be described as a function of the dose at various levels, including single/multiple neurons, clusters, regions, or networks. Here, we are focused on the received stimulation dose obtained from computational head models and brain responses which are quantified by functional magnetic resonance imaging (fMRI) data. In this randomized, triple-blind, sham-controlled clinical trial, we recruited sixty participants with methamphetamine use disorders (MUDs) as a sample clinical population who were randomly assigned to receive either sham or active tDCS. Structural and functional MRI data, including high-resolution T1 and T2-weighted MRI, resting-state functional MRI, and a methamphetamine cue-reactivity task fMRI, were acquired before and after tDCS. Individual head models were generated using the T1 and T2-weighted MRI data to simulate electric fields. In a linear approach, we investigated the associations between electric fields (received dose) and changes in brain function (response) at four different levels: voxel level, regional level (using atlas-based parcellation), cluster level (identifying active clusters), and network level (task-based functional connectivity). At the voxel level, regional level, and cluster level, no FDR-corrected significant correlation was observed between changes in functional activity and electric fields. However, at the network level, a significant positive correlation was found between frontoparietal connectivity and the electric field at the frontopolar stimulation site (r = 0.42, p corrected = 0.02; medium effect size). Our proposed pipeline offers a methodological framework for analyzing tDCS effects by exploring dose-response relationships at different levels, enabling a direct link between electric field variability and the neural response to tDCS. The results indicate that network-based analysis provides valuable insights into the dependency of tDCS neuromodulatory effects on the individual’s regional current dose. Integration of dose-response relationships can inform dose optimization, customization, or the extraction of predictive/treatment-response biomarkers in future brain stimulation studies.

**Summary**

In this study, we sought to understand how varying received doses of transcranial direct current stimulation (tDCS), a non-invasive brain stimulation technique, influence brain function as measured by functional magnetic resonance imaging (fMRI). We explored this "dose-response" relationship in participants with methamphetamine use disorders, using them as a representative clinical sample. Leveraging computational models based on individual MRI scans, we predicted cortical electric fields (EFs) as indicators of the received stimulation dose for each participant. Our study aimed to uncover the relationship between these EFs and changes in brain function at various levels: from individual brain locations (voxels) to broader network connections. While no significant correlation emerged between EFs and brain activity when focusing on individual voxels, specific regions of interest, or clusters, a notable correlation was observed at the broader network level, particularly between EFs and the frontoparietal connectivity during drug cue exposure. These results suggest that the effects of tDCS might be more effectively understood by examining wider brain networks rather than just individual points or regions. This research sheds light on the potential of individualized treatment plans using tDCS, underscoring the significance of comprehending its impact on interconnected brain networks.

## 1. Introduction

Non-invasive brain stimulation techniques, particularly transcranial direct current stimulation (tDCS), have gained prominence in investigating the relationship between modulated brain regions and stimulation outcomes [1,2]. tDCS has shown promising results in modulating brain activity and connectivity in both healthy individuals and those with neurological or psychiatric disorders [3–5]. Previous studies have demonstrated that tDCS can alter cortical excitability and brain functions [2,6]. However, the inter-individual variability of tDCS poses challenges in detecting intervention effects at the group level [7], and small effect sizes further limit its potential efficacy [8–10].

The variability in tDCS responses can be attributed to anatomical and functional factors, including individual differences in fat thickness, skull thickness, cerebrospinal fluid volume, ongoing brain activity, and connectivity [7,11–13]. Computational models are commonly employed to estimate the received stimulation dose, as it is not feasible to directly measure cortical electric field (EF) strength invasively [14]. These models have been validated through intracranial recordings, physiological measurements, and neuroimaging studies [15–19]; (e.g., see [20,21] for more details on SimNIBS validation and its comparison with other computational modeling method which are commonly used for creating computational head models). Computational head models based on high-resolution structural MRI provide insights into the distribution of EFs across the cortex, with the assumption that EF intensity relates to functional responses at the cortical target site. Investigating the dose-response relationship between the EFs and underlying brain functions can thus help explain the effects of tDCS [22].

To investigate the dose-response relationship and understand how tDCS-induced EFs influence brain functions, multiple modalities of magnetic resonance imaging (MRI) data, including structural and functional MRI, could be informative. Structural MRI is used to create individualized computational head models that predict the distribution patterns of EFs over the cortex. Functional MRI captures cortical functional activity in response to the injected current. Few studies have integrated computational head models with functional MRI data to explore dose-response relationships.

Based on our systematic review, as of the end of Jun 2023, only twelve studies have integrated computational head models with fMRI data to measure dose-response associations in both healthy and clinical populations [1,22–32]. Available dose-response studies could be categorized into two main approaches: regional-based and network-based methods that used a simple linear correlation/regression analysis to quantify dose-response relationships. Previous investigations of dose-response relationships have primarily focused on regional-based association which has been performed at different levels including voxel-level, region-of-interest (ROI)-level, or cluster-level, and some significant (e.g., [1]) or non-significant associations (e.g., [32]) were also reported. However, brain regions are functionally connected, and exploring the relationships between dose (EFs) and response (brain functions measured with fMRI) within brain circuits or networks is crucial. Three published papers have explored the dose-response at the network-level [26,31,32]. For example, it has been shown that current density in the left DLPFC positively correlated with changes in functional connectivity between two predefined ROIs (left dorsolateral and left ventrolateral PFC) during a working memory task, using psychophysiological interaction analysis and simulating precise computational head models [26]. Despite the available evidence according to previously published papers, there is no clue about which level of integration from voxel to network would be more informative in dose-response relationship analysis based on integrating head models with fMRI data.

This study aimed to examine the association between individualized received dose (EFs estimated using subject-specific computational head models) and brain response (changes in fMRI data) at multiple levels. Although these four levels of analysis are interconnected, we explored all four levels to determine which approach may be more effective for future dose-response analyses. By investigating the associations at different levels, we aimed to gain a comprehensive understanding of the dose-response relationship in tDCS and identify the most informative pipeline for studying the effects of stimulation. This analysis allows us to evaluate the relative strengths and limitations of each level, providing insights into which level of analysis may be more effective in elucidating the underlying mechanisms of tDCS-induced brain modulation. Given the highly interconnected nature of the brain, we hypothesize that associations between EFs and functional changes will be stronger at the network level compared to other levels of analysis. Here, we present the data from our pre-registered trial (NCT03382379) [33], which investigated the dose-response relationship in a group of individuals with methamphetamine use disorders (MUDs) as a sample of a clinical population offering novel insights into the effects of tDCS and its potential application in personalized treatment approaches.

## 2. Method

### 2.1. Ethics statement

This study was approved by the Western Institutional Review Board (WIRB Protocol #20171742, Transcranial Direct Current Stimulation to Modulate Top-Down Regulation for Drug Craving in Methamphetamine Use Disorder [NeuroMethDC]). The research adhered to the principles of the Declaration of Helsinki and was conducted in compliance with relevant guidelines and regulations. Formal written consent was obtained from all participants before any procedures took place. All participants were adults; thus, no parental or guardian consent was required.

### 2.2. Participants

Participants included 60 individuals (all-male, mean age ± standard deviation (SD) = 35.86 ± 8.47 years, ranging from 20 to 55) diagnosed with methamphetamine use disorder (MUD). They were recruited during early abstinence from the 12&12 residential drug addiction treatment center in Tulsa, Oklahoma as part of a clinical trial investigating the effectiveness of tDCS in reducing methamphetamine craving (ClinicalTrials.gov Identifier: NCT03382379). Further details regarding the inclusion and exclusion criteria can be found in the supplementary materials S1 Text.

### 2.3. Data collection procedure

**Fig 1:**
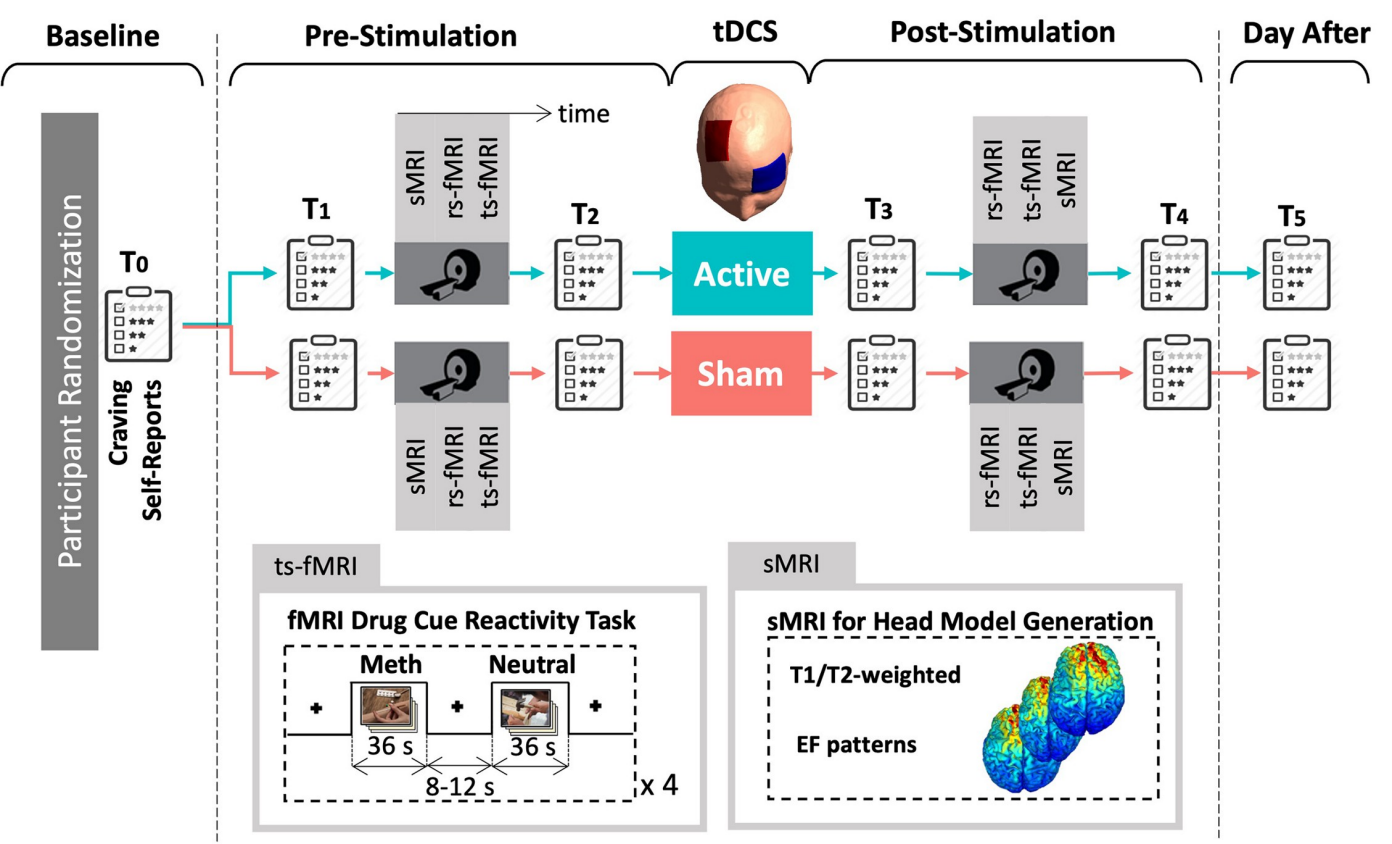
Data acquisition procedure. 60 participants with methamphetamine use disorders were randomly assigned to the active or sham group. MRI data including structural MRI for creating head models, resting-state fMRI, and fMRI drug cue reactivity task were collected before and after unilateral DLPFC stimulation. Behavioral data including VAS scores were also collected immediately before and after MRI sessions, at the baseline, and the day after. Abbreviations: sMRI: structural MRI, rs-fMRI: resting-state fMRI, ts-fMRI: task-based fMRI, EF: electric field.

The data acquisition procedure for this study is depicted in Fig 1. It was a randomized, triple-blind, sham-controlled clinical trial with two parallel arms. Participants provided written consent and were randomly assigned to receive either active or sham stimulation. Demographic and substance use profiles of each group and the primary outcomes of the trials are presented in our previously published paper [33]. Neuroimaging data, including structural MRI, resting-state, and task-based fMRI, were collected in a pre-stimulation/post-stimulation design. Methamphetamine craving was assessed using a Visual Analogue Scale (VAS) at multiple time points.

The methamphetamine cue reactivity (MCR) task was conducted following each resting-state scan. The MCR task involved presenting pictorial methamphetamine cues in a block design, consisting of two sets of distinct but equivalent pictures that had been validated in a previous study. The total duration of the task was approximately 6.5 minutes and included 4 blocks of neutral pictures and 4 blocks of methamphetamine pictures. Each block consisted of 6 pictures from the same category (meth or neutral), with each picture presented for 5 seconds and a 0.2-second blank interval between them. A visual fixation point was displayed for 8 to 12 seconds between each block. After each MRI session participants were asked to rate their current level of methamphetamine craving on a scale from 1 to 100 using VAS score. Craving scores were also collected at the baseline session and the day after the stimulation. More details on imaging parameters can be found in supplementary materials S2 Text.

### 2.4. Transcranial direct current stimulation

tDCS was administered using two saline-soaked surface sponge electrodes, each with an area of 5x7 cm2, connected to the battery-driven NeuroConn DC stimulator MR. To target the right DLPFC unilaterally, a bipolar non-balanced configuration was employed based on "Beam F3-system" [34] by taking into account the nasion-to-inion and tragus-to-tragus distances, as well as head circumference, to determine the electrode coordinates for each individual’s scalp. The anode electrode was positioned over F4 in the EEG 10–20 standard system, with the long axis of the pad pointing towards the vertex of the head. The cathode electrode was placed over the contralateral eyebrow (Fp1 EEG electrode site, also known as the supraorbital position), with the long axis of the pad parallel to the horizontal plane. To secure the electrodes in place, multiple rubber headbands were used.

During active stimulation, tDCS was delivered for 20 minutes at an intensity of 2 mA, with a 30-second ramp-like fade-in, followed by 19 minutes of active stimulation, and a 30-second ramp-like fade-out. For the sham stimulation procedure, the stimulator automatically turned off after 100 seconds, consisting of a 30-second ramp-like fade-in, 40 seconds of active stimulation, and a 30-second ramp-like fade-out. This simulated the typical sensations induced by tDCS. In the sham group, the fade-out was followed by 18.3 minutes without any further stimulation, during which impedance was periodically checked to ensure the average current over time did not exceed 2 μA. Throughout both active and sham tDCS sessions, the impedance was maintained below 10 k-Ohm.

### 2.5. fMRI data preprocessing

Functional data analysis was conducted using the AFNI software package. Preprocessing steps were applied, including the removal of the first three pre-steady state images. The following procedures were performed: despiking, slice timing correction, realignment, transformation to MNI space, and Gaussian FWHM smoothing with a kernel size of 4 mm. To account for potential confounding factors, three polynomial terms and the six motion parameters were regressed out during the analysis. Additionally, TRs with excessive motion were identified and censored based on a criterion defined as the Euclidean norm of the derivative of the six motion parameters exceeding 0.3.

The modeling of the neutral and methamphetamine cue blocks was accomplished by convolving the stimulus timing with a 31-second block regressor, resulting in the estimation of one response for each condition before and after stimulation. Notably, the primary analysis did not incorporate separate regressors for box trials or response periods.

### 2.6. Computational head modeling

High-resolution T1-weighted MR images were used along with SimNIBS 3.1 software to generate personalized computational head models [20]. The head models consisted of six main tissues: white matter (WM), gray matter (GM), cerebrospinal fluid (CSF), skull, vitreous bodies of the eyes, and skin. The segmentation process was performed using the "headreco" function in SPM 12 combined with the CAT12 toolbox, and the accuracy of the segmentations was visually inspected against the high-resolution T1-weighted MR images. Tetrahedral volume meshes were created based on the segmented surfaces, with approximately 3 million tetrahedra assigned to each personalized head model. Virtual rectangular pads with dimensions of 5 x 7 and a thickness of 1 mm were modeled and placed on the scalp of each realistic head model, corresponding to the anode and cathode positions over F4 and Fp1, respectively. The conductivity values used for the different tissues in the simulations were based on previously reported values: WM = 0.126 S/m, GM = 0.275 S/m, CSF = 1.654 S/m, skull = 0.010 S/m, skin = 0.465 S/m, and eyeballs = 0.5 S/m [32]. To simulate the distribution patterns of the electric field, a current strength of 2 mA was applied, and the electric field (EF = -∇φ) was solved using a finite element solver (FEM) under the assumption of a quasi-static regime [35]. The tangential electric field, representing the strength of the electric field, and the radial electric field, reflecting the currents entering or leaving the cortex, were calculated for each individual. The simulation results were then transformed into the standard space to enable comparability for group-level analysis. The visualization of the results was performed using Gmsh [36] and MATLAB (version 2019b, The Math Works Inc). General findings about EF simulations can be found in supplementary materials S3 Text.

While SimNIBS is a widely used method for creating computational head models in brain stimulation studies and validated before [20,37,38], it is worth mentioning that there are alternative methods available for indirectly estimating tDCS-induced electric field strength using MRI imaging. One such method is MR-based current density imaging (CDI), which allows for the monitoring of current flow inside the brain during tDCS [39–41]. CDI involves generating personalized three-dimensional volume conductor models using anatomical MR images and incorporating directional information from diffusion tensor MRI scans (DT-MRI) along with measured magnetic flux density data resulting from the externally applied stimulation current. By assuming an anisotropic conductivity distribution, the method calculates a model-predicted current density and magnetic flux density distribution. Through an iterative process that compares the differences between measured and computed magnetic flux density data, the transversal components of the current density distribution produced by the stimulation current are updated. The CDI technique has been validated using realistic three-dimensional human head models, measured DT-MRI images, and simulated magnetic flux density data, providing reliable and quantitative visualization of internal current density distributions during tDCS [39,40].

### 2.7. Association analysis

**Fig 2:**
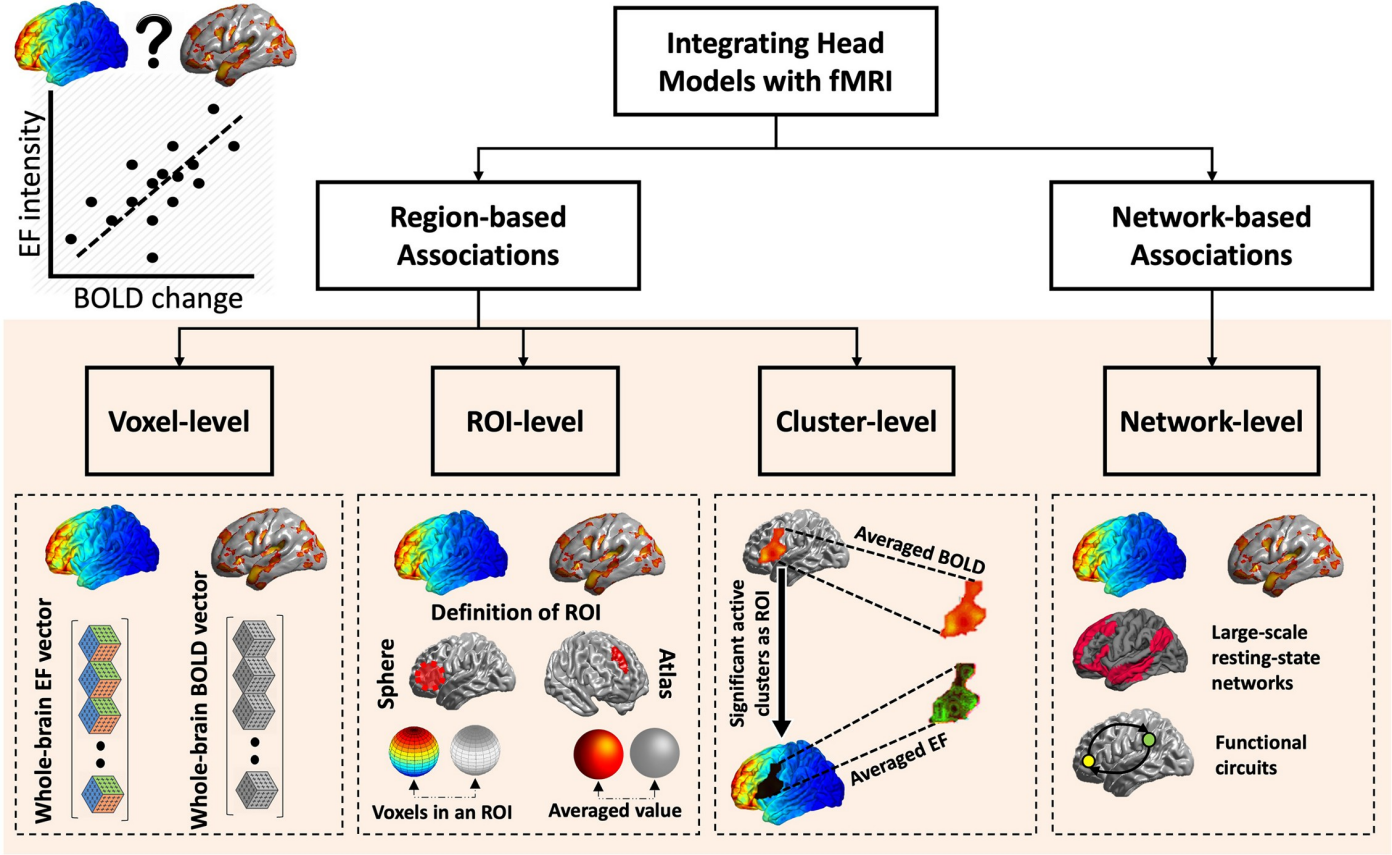
Framework for Data Analysis. The integration of computational head models with fMRI data was achieved through regional-based associations and network-based associations. Regional-based methods encompassed three levels: voxel-level, regions of interest (ROIs)-level, and cluster-level, while network-based associations involved exploring the relationships between electric fields (EF) and changes in functional activity/connectivity within large-scale networks or functional circuits.

A sample size of 30 participants per arm provided 80% power to detect an effect size (Cohen’s d) of 0.74. Associations were investigated using Pearson Correlation Coefficients with FDR correction (q was set to 5%). The associations between cortical electric fields (received dose) and changes in brain function (response) were examined at four different linear levels (Fig 2 is a schematic representation of each level). (1) Voxel-level associations: Analyzing correlations between EFs and fMRI data at the level of individual voxels. Each voxel represents a small three-dimensional element in the brain which includes a complex mixture of neural and non-neural components like neurons, glial cells, blood vessels, and interstitial fluid. (2) ROI-level associations: Assessing changes in brain activation during the drug cue reactivity task by calculating average measures of activity within specific regions of interest (ROIs). ROIs are collections of voxels that are defined based on anatomical criteria such as atlas based parcellation. (3) Cluster-level association: Investigating the effects of tDCS on whole-brain functional activation by identifying clusters of contiguous voxels that exhibit significant changes in activity. A cluster represents a group of adjacent voxels with similar functional characteristics. (4) Task-based network-level associations: Examining patterns of functional connectivity between a seed region and the rest of the brain or between two seeds during the drug cue reactivity task. Functional connectivity refers to the temporal correlation of activity between different brain regions, and a network represents a set of interconnected regions. Association analyses were performed in the active group. However, due to significant results at the network-level we also checked if there is any significant correlation in the sham group at the network-level.

### 2.8. Regional-based associations

#### 2.8.1. Voxel-level associations

A block design analysis was conducted on the meth > neutral contrast in pre- and post-stimulation scanning sessions using general linear models (GLM). The functional map and computational head models were transformed to MNI space, excluding non-brain voxels. EFs were resampled to match the resolution of the functional map, resulting in final maps comprising approximately 1050624 (96 x 114 x 96) voxels. Three-dimensional voxels were vectorized, along with the different columns of a matrix for each map, where participants were stacked along the rows. Let Xi and Yi represent the column vectors across all participants for the i-th voxel from the EF and fMRI matrices. A whole-brain voxel-wise correlation was calculated between each corresponding voxel within the brain. The spatial distribution of the correlations (r-values and p-values) was then used to reconstruct the brain map. Statistical results were corrected for multiple comparisons using false discovery rate correction (FDR).

#### 2.8.2. ROI-level associations

For ROI-level analysis, brain activation during the drug cue reactivity task was calculated for the meth > neutral contrast before and after stimulation separately. Mean beta weight values were estimated for all extracted ROIs using the Brainnetome atlas parcellation [42] to assess the level of changes in task-related activity during the meth versus neutral condition. Brainnetome atlas was also applied to computational head models in the active groups and averaged EF strength was extracted from each subregion. To determine the relationship between induced EF in each brain region and changes in neural activation (i.e., functional activity after tDCS compared to before stimulation) in response to cue exposure, the correlation between averaged EF strength and changes in functional activity was calculated in each corresponding Brainnetome atlas sub-regions. Then, FDR correction was applied to the statistical results.

#### 2.8.3. Cluster-level association

The effects of tDCS on whole-brain functional activation were studied using a linear mixed-effect model (LME) at the voxel-level, which accounted for the non-independence of observations in repeated measure data. The model included time (pre- and post-stimulation) and group (sham and active), along with their interaction, as fixed effects. The subject was treated as a random effect. The lme function of the R-package nlme was implemented to fit the linear mixed model [43]. To identify clusters showing significant changes in functional activation, a cluster-based analysis was performed. This analysis considered both regions of increased activation and regions of decreased activity following tDCS intervention. The group-level functional t-map was computed in MNI space, and family-wise error (FWE) correction was applied using Monte Carlo simulations (3dClustSim, AFNI) with an alpha threshold > 0.1. Results with a significance level of P < 0.005 and cluster size > 40 were considered statistically significant.

For each identified cluster, the averaged BOLD signals were extracted, representing the combined response within the cluster. Cluster masks were then applied to the computational head models to extract the averaged EF intensity from each cluster. Correlations between EFs and the functional map obtained from the LME model were calculated at the cluster level using Pearson Correlation Coefficient analysis. In this study, a cluster-based analysis was employed to offer a comprehensive assessment of the functional changes induced by transcranial direct current stimulation (tDCS). This analytical approach is designed to capture both regions of increased and decreased neural activation, thereby providing a holistic view of tDCS effects on the brain. Importantly, the cluster-based method allows for the detection of distributed effects across various brain regions, encapsulating both positive and negative modulations in functional activity and connectivity. The study specifically focused on temporal changes (pre- vs. post-stimulation) and the interaction between time and experimental groups (active vs. sham) to isolate the effects attributable to the tDCS intervention. While baseline differences between the active and sham groups were possible, these were rigorously accounted for in post hoc analyses to ensure that the observed functional changes were indeed a consequence of the tDCS intervention rather than pre-existing group differences.

### 2.9. Network-based associations

#### 2.9.1. Task-based network-level associations

To examine seed-to-whole-brain task-based functional connectivity, a seed region was defined based on the location of the maximum EF. The individualized computational head models were transformed into fsaverage space to calculate the averaged EF across the entire population. From the averaged EF map, the 99^th^ percentile of the maximum EF was determined, and a 10 mm sphere was defined around this threshold. This sphere was used as the seed region for conducting generalized psychophysiological interaction (gPPI) analysis in CONN toolbox [44], which identifies voxels in the brain exhibiting altered connectivity with the seed region during a specific context, such as the drug cue reactivity task in this study. The averaged BOLD signal was extracted from the seed region, and task-modulated functional connectivity was computed. At the first-level analysis, the design matrix included the time course of the seed region, the cue exposure task time course, and the interaction between the task and the BOLD signal in the seed region. For the second-level analysis, an interaction between time (pre- vs. post-stimulation) and group (active vs. sham) was calculated. The voxel-level threshold was set at p uncorrected < 0.01, and the cluster-level threshold was set at p FDR corrected < 0.05. To consider the significant clusters obtained from the seed-to-whole PPI results, the masks of these clusters were utilized. ROI-to-ROI PPI connectivity analyses were then performed between the seed region located around the 99^th^ percentile and the cluster masks. The correlation between ROI-to-ROI PPI connectivity and averaged EFs in the seed region was calculated in each active and sham group separately.

### 2.10. Considering behavioral data

Behavioral data, specifically VAS scores obtained through the question *’How much craving do you have right now*?’, were collected immediately before and after each MRI session. Significant associations were observed between EFs and BOLD signal at the network-level, indicating a connection between the measured neural activity and the distribution patterns of EFs. To further investigate the relationship between the obtained results and behavioral outcomes in both the active and sham groups, additional correlations were computed between VAS scores, BOLD signal, and EFs.

## 3. Results

### 3.1. Regional-based associations

#### 3.1.1. Voxel-level results

**Fig 3:**
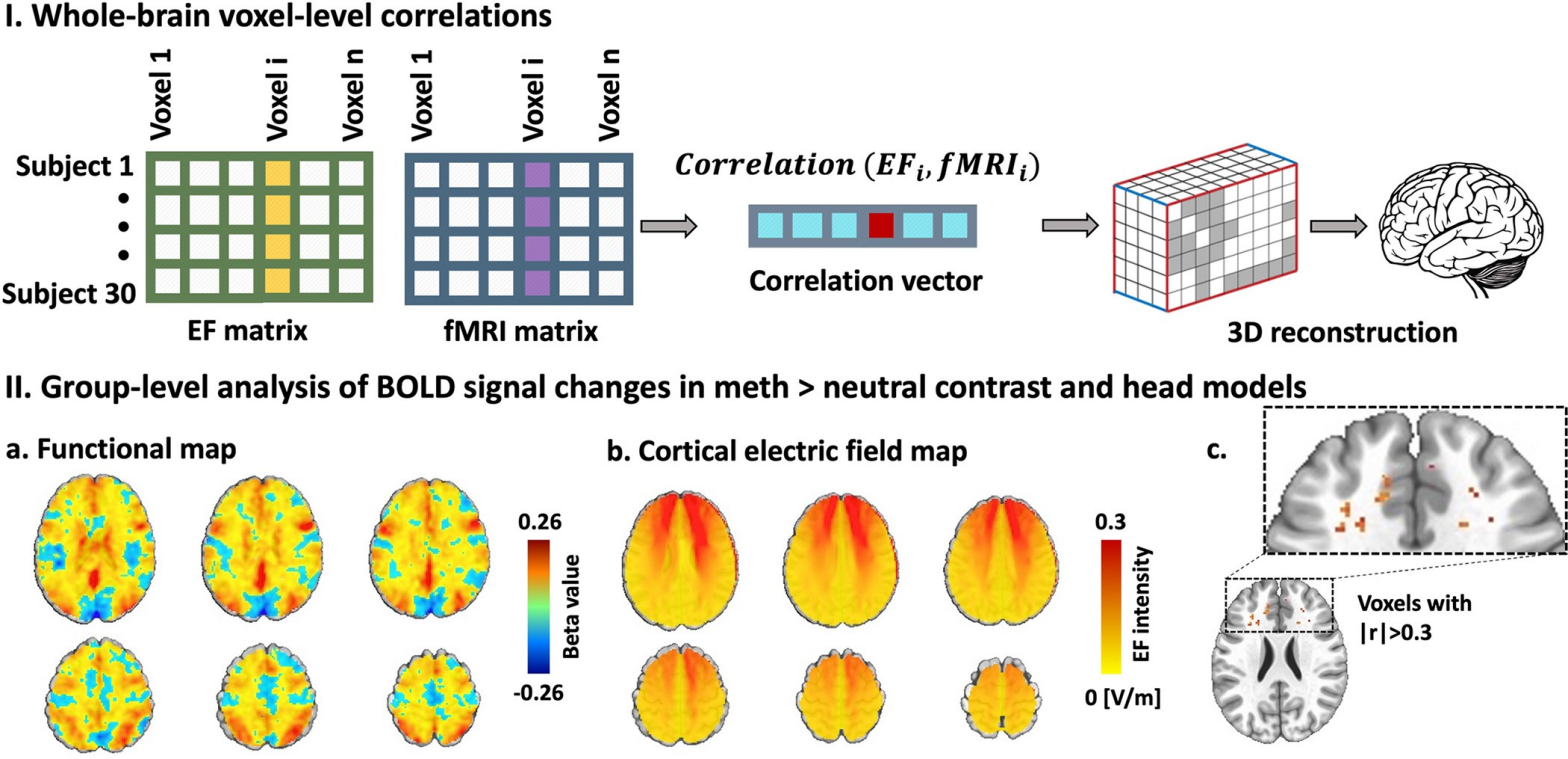
Voxel-level associations. **I. Whole-brain voxel-level correlation analysis pipeline.** Dose-Response (EF-fMRI correlation) at the voxel-level in a whole-brain approach. a. Electric field (EF) maps were vectorized and arranged along the columns, with participants in the active group represented along the rows to construct the EF matrix. A similar approach was employed to generate an fMRI matrix for the meth > neutral contrast. The active group comprised 30 participants, and the total number of voxels analyzed was 96 x 114 x 96. The correlation vector captures the relationship between each corresponding voxel in the EF and fMRI matrices. II. Whole-brain voxel-level correlation analysis results. a. Group-level functional map. Changes in BOLD signal (Post–Pre) in the meth vs. neutral contrast at the group-level for participants in the active group. b. Group-level analysis of head models. EF distribution patterns observed in participants in the active group, with anode/cathode positioned over F4/Fp1. c. Correlation results. Prior to the false discovery rate (FDR) correction, some voxels showed |r|>0.3 and p uncorrected< 0.05. However, none of them survived FDR correction.

At the whole-brain voxel-level, block-designed analyses revealed no FDR-corrected significant correlation between electric fields and BOLD signal changes (p corrected > 0.05). Only a small proportion of voxels exhibited a small effect size (0.1 < |r| < 0.3) (9.74% of the voxels), a medium effect size (0.3 < |r| < 0.5) (1.36% of the voxels), and a mere 0.09% of voxels showed a large effect size (0.5 < |r| < 1). The total number of voxels analyzed was 96x114x96, and the Pearson Correlation Coefficient (|r|) was used as a measure of effect size (Fig 3).

#### 3.1.2. ROI-level results

**Fig 4:**
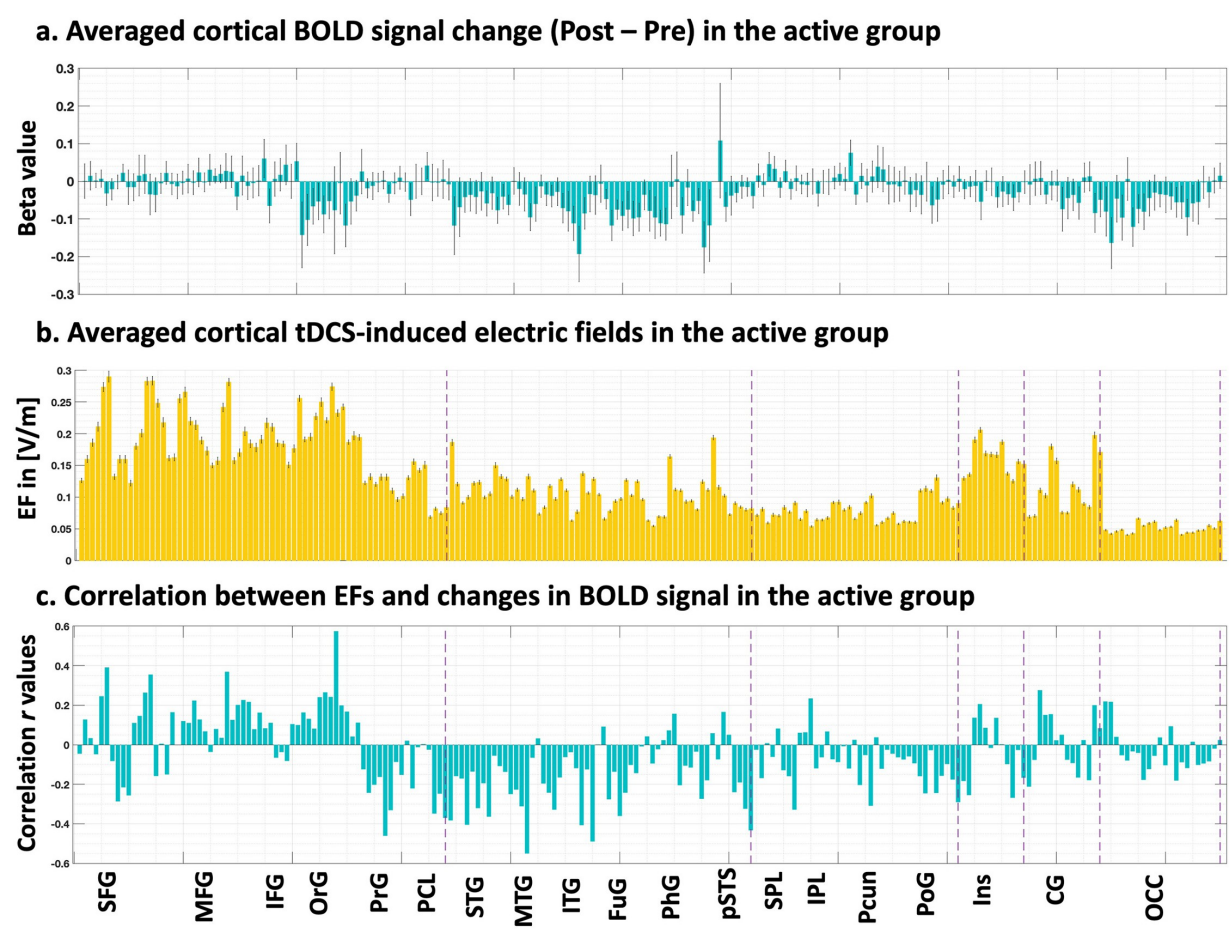
ROI-level associations. **a. Cortical BOLD signal change.** Applying Brainnetome atlas parcellation to the first-level functional maps obtained from general linear models (Post minus pre-stimulation and meth vs. neutral contrast) for the active group. **b. Individualized computational head models.** Head models were parcellated using the Brainnetome atlas. Averaged electric fields (EFs) were calculated only in cortical subregions. **c. Correlation r values.** Pearson correlation coefficients between all pairs of ROIs (EFs and BOLD signal change) were calculated in the active group and none of them survived FDR correction. Bars show mean value and error bars show standard errors.

At the ROI-level, pairwise correlations between EFs and changes in fMRI BOLD signal (Post–Pre) was conducted in the meth vs. neutral contrast, using atlas-based parcellation of individualized head models and fMRI maps with the Brainnetome atlas (Fig 4). At the whole-brain ROI-level, results revealed no FDR-corrected significant correlation between EFs and BOLD changes considering p corrected > 0.05. However, without FDR correction, we identified 11 ROIs that showed a correlation with p uncorrected < 0.05. Among these ROIs, a small proportion exhibited a medium effect size (0.3 < |r| < 0.5), accounting for 4.29% of the total ROIs analyzed, while only 0.95% of the ROIs displayed a large effect size (0.5 < |r| < 1) including two brain regions Brodmann area 11 medial area located in orbital gyrus (r = 0.58, p uncorrected = 0.001) and Brodmann area 21 rostral area in middle temporal gyrus (r = -0.55, p uncorrected = 0.002). Only the first region received EFs above 0.2 V/m, however, did not survive FDR correction.

#### 3.1.3. Cluster-level results

**Fig 5:**
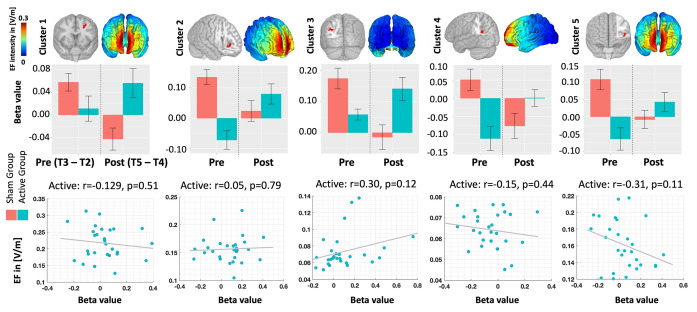
Cluster-level associations. Top row: Clusters exhibiting significant time-by-group interactions in the second-level analysis of individualized fMRI maps. Group-level computational head models are also depicted in the corresponding view. Middle row: BOLD signal within the identified significant clusters, represented separately for each group in Pre- and Post-stimulation conditions. Bottom row: Correlation between BOLD signal and EFs within the clusters. Notably, no significant correlation was observed at the cluster level. However, the regression analysis revealed a significant effect for the group. Bars show mean value and error bars show standard deviations.

Five active clusters in the left hemisphere surpassed our predefined threshold without undergoing multiple error comparison corrections (voxel-level threshold: p uncorrected < 0.005, cluster size > 40). These clusters encompassed the middle frontal gyrus (Cluster 1), anterior insula (Cluster 2), inferior parietal lobule (Cluster 3), precuneus (Cluster 4), and inferior frontal gyrus (Cluster 5). Upon analyzing these clusters at the cluster-level (see Fig 5), our findings revealed no significant correlation between changes in functional activity and EFs within these clusters (p uncorrected > 0.05). Among the clusters examined, 80% exhibited small effect sizes.

### 3.2. Network-based association

#### 3.2.1. Network-level results

**Fig 6:**
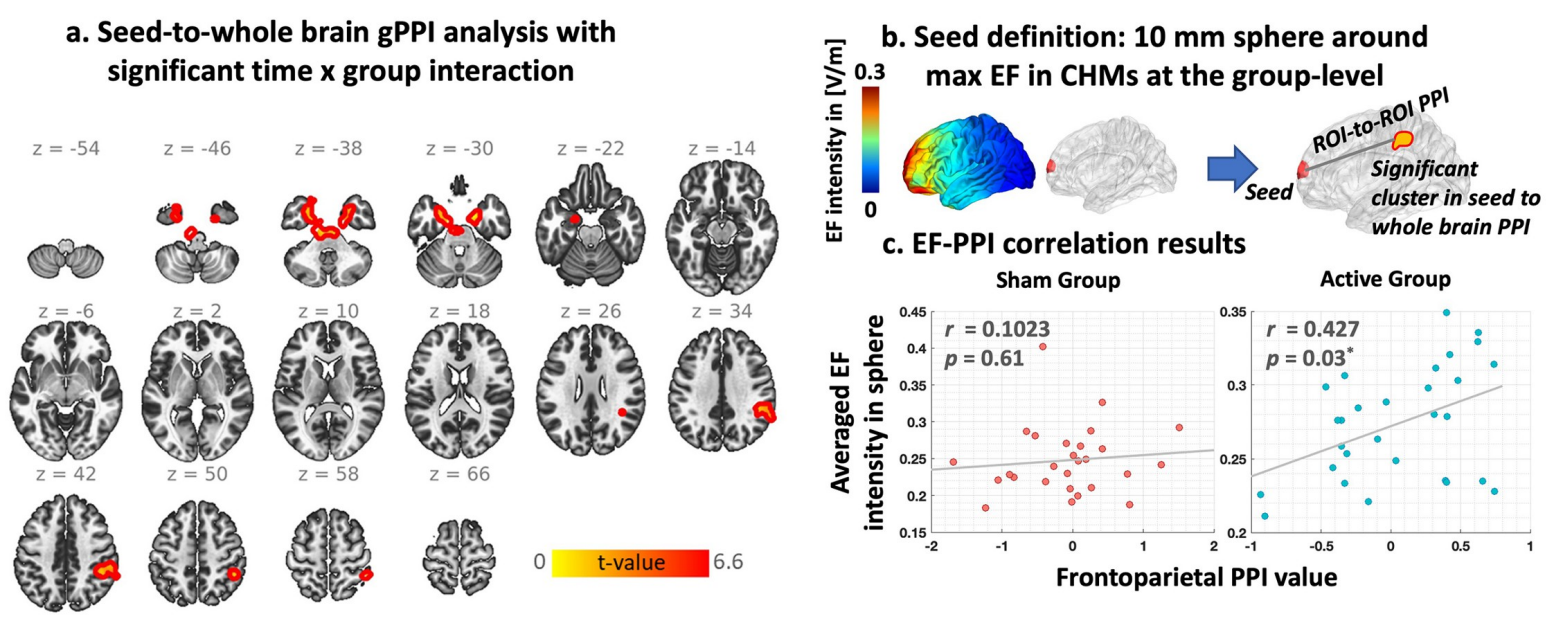
Dose-Response (EF-fMRI correlation) at the Network-level integration based on seed-to-whole brain generalized psychophysiological interaction (gPPI). **a.** Brain regions with significant time by group interaction in gPPI analysis (two significant clusters in bilateral amygdala and hippocamp, and right inferior parietal lobule (IPL)). **b.** defining seed region based on a 10 mm sphere around the 99^th^ percentile of the electric field (EF) location. **c.** correlation between averaged EF intensity within the 10 mm sphere and PPI connectivity between the sphere around the 99^th^ percentile of the EFs and IPL cluster in each group separately (active: r = 0.42, P corrected = 0.03, sham: r = 0.10, p corrected = 0.61).

Our gPPI results (Fig 6) showed two significant clusters as follows: (1) located in bilateral amygdala and hippocamp (cluster size: 1356, (x, y, z) peak coordinate in MNI space: (18,-2,-30) and t-value: 6.59), and (2) located in right inferior parietal lobule (IPL) (cluster size: 701, (x, y, z) peak coordinate in MNI space: (46,-38,38), and t-value: 5.20). ROI-to-ROI gPPI connectivity was also calculated between seed region and each cluster and correlation between averaged EF in seed region and seed-to-cluster gPPI connectivity was calculated. For the network-level analysis, frontoparietal connectivity showed a positive significant correlation with EF in the frontal stimulation site (r = 0.43 (medium effect size), p corrected = 0.03). We also checked the correlation results between simulated EFs and frontoparietal task-based connectivity in the sham group and no significant correlation was found (r = 0.10, p corrected = 0.61).

### 3.3. Behavioral results and correlation with EF and neural response at the network-level

Participants rated their cravings based on the Visual Analog Scale (VAS) ranging from 0 to 100 in both active and sham stimulation groups. The linear mixed-effects (LME) analysis revealed a significant effect of time (P < 0.0001), indicating changes in craving scores over time. However, there was no statistically significant effect of group or time × group interaction (Fig A in S4 Text).

During the pre-stimulation phase, both the active and sham groups showed a significant increase in craving scores (P uncorrected < 0.0001 for both groups) after the fMRI cue-induced craving task in the first scan (T2 time point in Fig 1) compared to the initial assessment (T1). The mean craving scores ± SE were 59.07 ± 6.17 for the sham group and 64.40 ± 5.20 for the active group at T2, while at T1, the scores were 39.4 ± 6.07 for the sham group and 42.50 ± 5.26 for the active group. Our results demonstrated that there was no significant increase (P uncorrected > 0.05) in self-reported craving after the second exposure to drug cues (T4) compared to before the second cue exposure (T3). The mean craving scores ± SE were 24.67 ± 4.96 for the sham group and 27.23 ± 4.63 for the active group at T4, while at T3, the scores were 17.83 ± 21.55 for the sham group and 25.93 ± 3.77 for the active group. Furthermore, a significant reduction in craving scores (P uncorrected < 0.05 for both groups) was observed after stimulation (T3) compared to the before stimulation (T2) for both the sham and active groups. The mean craving scores ± SE were 17.83 ± 21.55 for the sham group and 25.93 ± 3.77 for the active group at T3, while at T2, the scores were 59.07 ± 6.17 for the sham group and 64.40 ± 5.20 for the active group. Additionally, a significant difference in craving scores was found between the baseline (T0) and the day after stimulation (T0: sham 49.37 ± 5.74, active 61.37 ± 4.50; T5: sham 11.17 ± 3.60, active 12.67 ± 3.33).

Despite the absence of significant differences between the active and sham groups in terms of craving self-reports, our exploration at the network level revealed significant correlations between electric fields (EFs) and neural responses only in the active group. To further investigate potential relationships between EFs, neural responses, and behavioral outcomes, we examined the correlation between behavioral data (changes in VAS scores) and neural responses or EFs at the network level. However, our analysis did not identify any significant correlations between changes in behavioral data and either frontoparietal PPI connectivity (meth vs. neutral) changes or EFs at the network level. We only found a significant correlation (r = 0.37, p uncorrected = 0.049) between frontoparietal PPI connectivity during meth cue exposure in the pre-stimulation scan (at first scan which can be named baseline) and changes in craving score (VAS changes from T3 to T4 which can be named second cue-induced craving) after active stimulation.

## 4. Discussion

In this study, we explored the impact of electric fields (EFs) on brain function, assessed through fMRI, across four analytical levels: voxel, ROI, cluster, and network. Voxel and ROI-level analyses showed no significant correlation between EFs and BOLD signal changes after multiple comparisons correction. Cluster-level analysis identified five active clusters in the left hemisphere with a time-by-group interaction effect in response to tDCS, but these did not correlate with EFs. Network-level analysis, however, revealed significant correlation between the frontal stimulation site and frontoparietal networks, specific to the active stimulation group. Despite these neural changes, no significant differences in self-reported craving scores were observed between the active and sham groups. Finally, we found a significant relationship between baseline frontoparietal connectivity and craving score changes post-stimulation, but this did not correlate with EF-induced network changes, suggesting that EF effects on neural activity may not directly influence subjective craving levels. Taken together, our findings highlight the complexity of EF effects on neural and behavioral outcomes, underscoring the need for multi-level analyses to fully understand the impact of electrical stimulation interventions.

### 4.1. Voxel-level integration

The voxel-level analysis in our study did not reveal any significant correlation between EFs and changes in BOLD signal, which aligns with our initial hypothesis. In contrast, the only previous study that investigated the correlation between EFs and fMRI at the voxel-level reported a significant association between EF distribution patterns in a standard brain and a group-level functional map using a voxel-level approach [27]. It is important to note that the methodology employed in that previous study differed from ours. They calculated a single correlation across all voxels using a standard head model in MNI space and a group-level functional map derived from second-level analysis of fMRI data [27]. In our study, we considered individualized EF and BOLD signals for each participant separately and then calculated the correlation for each single voxel across the population in the active group. These methodological distinctions may contribute to the observed discrepancy in findings. Furthermore, it is worth mentioning that our results focused on BOLD signal changes, while the previous study reported the correlation using cerebral blood flow (CBF) data [27].

The interpretation of voxel-level results in the context of transcranial electrical stimulation poses several challenges. One important consideration is the spatial alignment of the EF and BOLD signal maps. For meaningful correlation or regression analyses, it is crucial to ensure that both maps are in the same space, either subject space or an averaged standard space, with the same resolution [30]. This alignment enables the examination of associations between corresponding voxels across the population. Furthermore, voxel-wise integration faces limitations due to the inherently low signal-to-noise ratio (SNR) of individual voxel data, which can impact the reliability of associations [45]. The large number of voxels involved in voxel-wise analysis also necessitates rigorous correction for multiple comparisons to avoid spurious findings. Consequently, the statistical power to detect significant associations at this level is generally low.

Despite the lack of significant correlations at the voxel level, it is important to consider the potential localized effects of EFs on neural activity. Although the overall voxel-level analysis did not reveal significant associations, a small proportion of voxels exhibited large effect sizes, suggesting that localized regions may respond more prominently to EF-induced effects. This localized influence may be attributed to the intricate interplay between stimulation parameters, neural circuitry, and individual differences in brain structure and function. Understanding the factors contributing to these localized effects can provide valuable insights into the optimization of transcranial electrical stimulation protocols and individualized treatment approaches. In summary, our voxel-level analysis did not identify significant correlations between EFs and BOLD signal changes across the entire cortex. However, the challenges inherent to voxel-wise analysis, including spatial alignment, SNR limitations, and the need for rigorous multiple comparison correction, should be considered when interpreting these findings. The identification of a small proportion of voxels with large effect sizes suggests potential localized effects of EFs on neural activity. Future studies should further investigate these localized effects and explore the factors influencing the variability in EF-induced responses across different brain regions.

### 4.2. ROI-level

At the ROI-level, we applied Brainnetome atlas parcellation to both individualized head models and functional maps in order to extract averaged EF intensity and beta values. While we observed strong correlations in specific brain regions, such as the orbital gyrus and middle temporal gyrus, none of these regions survived the false discovery rate (FDR) correction, as we considered all brain subregions in the Brainnetome atlas for our analyses. In this regard, previous studies have reported significant correlations within predefined regions of interest; however, there is a lack of consensus regarding rules or hypotheses for ROI selection. For instance, among studies that examined dose-response relationships at the ROI-level, some studies defined spherical seed regions with varying sizes, including 2.5 mm, 6 mm, 8 mm around specific coordinates such as the peak activation voxel in fMRI analysis results, mean center coordinates obtained from MRS analyses, or around the hand knob region [1,22,24]. The remaining studies utilized different types of atlas-based parcellations [23,46].

Despite the methodological differences, the ability to detect significant correlations at the ROI-level is highly dependent on how ROIs are defined. These definitions can vary widely, from using standard anatomical or functional atlases for parcellation to placing small spheres around specific brain coordinates. The chosen method for defining ROIs plays a critical role in successfully identifying meaningful relationships between dose and neural response. For example, Esmaielpour et al. employed two different approaches for ROI definition: anatomical atlas parcellation of the prefrontal cortex and parcellation based on EF strength [46]. Similar to our study, they extracted averaged EF strength and beta values in the general linear model (GLM) analysis of task-based fMRI data. They reported a single significant correlation in the frontopolar cortex, which exhibited the highest EFs among ten brain regions. However, no significant correlation was found based on anatomical atlas parcellation. This study underscored the importance of the parcellation method and ROI definition in dose-response relationship studies [46].

In dose-response relationships using regional-based approaches (including both ROIs or clusters), it is essential to extract features from each region. This involves averaging the estimated parameters, such as beta activation maps and EF intensities, across the voxels within the predefined ROIs to calculate the relationships between fMRI-derived parameters and transcranial electrical stimulation-induced EFs. The ROI-based approach overcomes the low SNR observed in voxel-wise analysis by aggregating data from multiple voxels. Moreover, performing statistical testing on ROIs rather than voxels reduces the number of tests conducted. However, this approach comes with certain limitations. Results in brain regions outside of the pre-defined and hypothesized areas may be overlooked, and the selection of appropriate brain regions can be challenging, requiring specific knowledge about the research problem for defining relevant areas.

Taken together, our ROI-level analysis demonstrated the absence of significant correlations between EFs and BOLD signal changes when considering multiple comparison corrections. This suggests that the effects of EFs may be region-specific and not uniformly distributed throughout the brain. The main challenge in finding significant correlations at the ROI-level corresponds to the absence of a hypothesis to appropriately define ROIs. Further investigations are needed to better understand the underlying mechanisms and functional implications of these region-specific effects. Additionally, future studies should carefully consider the methodology for defining ROIs and the potential trade-off between hypothesis-driven analysis and the risk of missing significant associations in unexpected brain regions.

### 4.3. Cluster-level

We did not find any significant correlation between EFs and changes in functional activity within the active clusters, despite the significant effect of group observed in all five clusters. Consistent with our findings, there is no previous study that reported cluster-based associations based on integrating head models with fMRI data. As we defined in Fig 2, all voxel-level, ROI-level, and cluster-level correlation is a kind of regional-based association. However, the cluster-based integration approach bridges the gap between voxel-level and ROI-level analyses by considering each voxel’s priority for integration while performing the analysis within contiguous clusters of voxels, similar to the ROI-based approach. However, several factors may contribute to cluster-level findings. First, it is possible that the complex relationship between EFs and neural responses within the identified clusters is not adequately captured by the cluster-level linear associates. The cluster-based integration approach relies on the aggregation of voxel-level data within a cluster, potentially averaging out subtle but significant effects present at the voxel level. Furthermore, the sensitivity and specificity of the cluster-level analysis may be influenced by factors such as the size and distribution of the clusters, as well as the statistical thresholds applied.

### 4.4. Network-level

In line with our hypothesis, significant dose-response relationships were observed at the network level, highlighting the importance of examining global network effects rather than local associations found in voxel-level, ROI-level, or cluster-level approaches. Specifically, we observed a significant positive correlation between task-based frontoparietal functional connectivity and EF intensity within the prefrontal cortex. These findings align with previous research conducted by Indahlastria et al. during a working memory task, where they reported a significant correlation between current density values in DLPFC and changes in task-based functional connectivity within the working memory network in older adults [26]. Another recent study also examined associations at the network-level and reported that the individualized electric field in cortical regions with regional EFs exceeding 99^th^ and 98.5^th^ percentile significantly predicts tES-induced outcomes in terms of functional connectivity within sensorimotor areas in the ipsilateral hemisphere in motor cortex stimulation of twenty-five chronic stroke individuals [31]. In another recent study [32], two different approaches were used for extracting EFs, to determine the highest EFs the mask included 1,000 gray matter voxels for which the highest EF values were estimated. Alternatively, to determine EFs in cortical regions that are related to the EEG alpha band activity, the mask included 1,000 occipital gray matter voxels with the strongest negative correlation between alpha amplitude and BOLD signal. They reported a significant correlation between EF in alpha-BOLD correlated region and changes in alpha amplitude, however, no significant correlation was observed between EFs and changes in functional connectivity within the default mode network during resting-state fMRI data among twenty-two healthy subjects [32].

Two main points should be highlighted based on our results. Firstly, our findings demonstrated significant associations exclusively at the network level, indicating that the neurobiological effects of tDCS may not manifest in a straightforward manner at the voxel, region, or cluster level. This emphasizes the significance of examining network-level changes during stimulation. In recent years, there has been a growing emphasis on network-based effects of brain stimulation technologies, and our results further support the notion that tDCS effects are likely to spread across networks rather than being confined to specific local brain regions. Our network-level analysis sheds light on the significant modulatory effects of EFs on functional connectivity within the brain. These findings support the growing emphasis on network-level changes during tDCS and provide insights into the complexity of EF-induced effects on network dynamics. Further investigations should explore the underlying mechanisms of these network-level effects and consider the broader implications for understanding the functional consequences of tDCS.

### 4.5. Considering SUDs as a clinical population

In this study, our specific focus was on individuals with methamphetamine use disorders (MUDs) as a representative sample of the clinical population. There are some other findings in terms of dose-response relationships in SUDs. For example, a pre-post tDCS-fMRI study with F4-F3 electrode montage (bilateral frontal with anode on the right) conducted on MUD participants revealed a significant correlation between changes in brain functions in response to drug cues and the averaged EF strength within the frontopolar area, as opposed to other prefrontal regions [46].

Moreover, our previous investigations have examined the impact of substance use disorders (SUDs) on electric field distribution patterns in relation to anatomical alterations [47]. Specifically, we focused on individuals with cannabis use disorders, comparing them to healthy controls within the context of tES studies, and reported significant structural alterations in participants with cannabis use disorders compared to healthy controls [47]. Additionally, studies involving transcranial magnetic stimulation (TMS) have explored the influence of such alterations and revealed lower gray matter volume in multiple areas of interest for TMS treatment associated with alcohol use disorders (AUDs) [48]. These findings suggest that alterations in brain structure due to SUDs may affect EF distribution patterns and stimulation outcomes as previous studies have shown that tES/TMS-evoked in cortical and subcortical activity depend on brain structure. Further investigations are needed to assess the generalizability of the results obtained in this study to healthy subjects [49].

### 4.6. General challenges in integrating head models with fMRI

Integrating EFs with fMRI data presents several challenges that impact result interpretation and accuracy. First, combining a static map (head model) with a dynamic map (fMRI) is difficult due to the complex interactions between targeted and non-targeted brain regions. This complexity makes it challenging to establish a direct relationship between EFs and changes in brain activity measured by BOLD signals. Interpreting the results of brain integration studies can be complex in part due to the intricate interconnections between brain regions. When analyzing the relationship between EFs and BOLD signals, it’s crucial to consider both positive and negative correlations. For instance, higher EF strengths might reduce functional connectivity, while lower strengths could enhance it. This complexity calls into question the specific mechanisms through which EFs influence brain networks. Additionally, it’s important to recognize that the commonly used statistical measures of connectivity lack a robust biophysical basis. While there are plausible reasons why higher EFs could reduce connectivity, such as the orientation of EFs and neurons, the concept of connectivity itself is largely statistical. Therefore, further research is needed to elucidate the biophysical foundations of connectivity and its interplay with EFs for a more nuanced understanding of brain stimulation outcomes.

Another challenge is selecting appropriate measures for EF and fMRI data, as different metrics provide distinct information about EF distribution patterns and brain activity. In addition to EF strength, the orientation of the EF plays a role in adjusting the "effective" intensity when it is not aligned with the axis from the dendrites to the axon of a neuron. It can also indirectly indicate which neurons are recruited, as those that are most aligned with the vector [50]. However, these considerations are all encompassed within the measurement of EF intensity. The accuracy of the modeling process for computational head models is crucial, as inaccurate modeling can affect the distribution of EFs. Precise tissue segmentation and conductivity assignment are essential for accurate computational head models [51–53]. The choice of integration space, whether subject space or standard space, also affects the results. While standard space integration allows for comparative analysis, using personalized models based on individual scans yields higher accuracy. Overcoming these challenges is vital to obtain reliable insights into the relationship between EFs and neural activity measured by fMRI. Future research should address these challenges and improve the integration process.

### 4.7. Limitations and future directions

While the current dose-response relationship studies exploring the associations between EFs and brain functions show promise, there are several areas that require further investigation. Integrating head models with fMRI is still a relatively new approach in tES studies, and although previous works have reported novel findings, replication studies are necessary to establish a comprehensive understanding of the relationship between EF distribution patterns and changes in BOLD signals.

The complexity of the underlying biophysical processes and the diverse nature of neural responses make it difficult to directly link electric field distribution patterns to the efficacy of tDCS. The predictive value of integrating EF distribution with fMRI data in determining treatment outcomes requires further investigation. To drive the field forward, several important steps should be considered. First, future research should focus on elucidating the biophysical mechanisms underlying the effects of EF distribution on neural responses [54–56]. This could involve combining computational modeling, animal studies, and advanced neuroimaging techniques to establish a more comprehensive understanding. Second, efforts should be made to develop standardized protocols and methodologies for integrating EF distribution and fMRI data as a measure of dose-response relationship [57]. This includes refining the methods for assessing and quantifying EF distribution, improving the sensitivity and specificity of fMRI measurements, and establishing consensus guidelines for data analysis and interpretation. Third, in this study, the sample size is relatively small. Additional investigation involving a larger sample size and more diverse participants (e.g., including females to study the potential role of sex differences and other types of substance use) is needed to generalize the results of this study. Larger-scale studies involving diverse populations and clinical conditions are needed to enhance the generalizability of the findings. By expanding the scope of the investigation, we can better assess the efficacy of tDCS interventions and identify potential predictors of treatment response.

Theoretically, the polarization of neuronal membranes correlates linearly with the electric field they’re exposed to [58–60]. However, this doesn’t mean that neurophysiological, behavioral, or clinical outcomes also follow a linear progression. Advancements in computational resources and machine learning techniques, such as deep learning, offer opportunities to explore more complex nonlinear and higher-order relationships between EFs and functional activity/connectivity at the subject or group level. For example, deep learning models can be trained using computational head models and fMRI data for classification or prediction purposes. Machine learning methods can also help identify responders and non-responders based on EFs in targeted and non-targeted brain regions, as well as the initial brain state. This approach may facilitate the identification of multi-modal biomarkers by leveraging the predictive role of baseline brain activation and individualized EFs.

The emergence of closed-loop tES-fMRI studies, where fMRI and tES are performed simultaneously, opens up exciting possibilities for real-time integration of personalized ongoing brain function with EFs [61]. This approach enables the optimization of stimulation parameters based on the interaction between EFs and neuronal activity. However, implementing closed-loop head model-fMRI integration in real-time poses challenges, as the online processing of computational head models and fMRI data is time-consuming [62]. Future studies will require high-speed algorithms and powerful computational tools to enable efficient implementation of closed-loop integration. In addition, future studies can consider integrating other modalities such as EEG, DTI, or behavioral data with computational head models and fMRI. Integrating multiple sources of information allows for the exploration of hybrid activation patterns, leading to the identification of putative trait-state relationships that could offer more informative insights than relying on a single data source.

## 5. Conclusion

In conclusion, this study investigated the integration of EFs with fMRI data and explored the associated challenges and implications. Overall, our findings underscore the complexity of the relationship between EFs, neural activity, and functional connectivity. The region-specific effects of EFs and their modulation of network-level connectivity provide valuable insights into the targeted and context-dependent nature of tDCS effects. Although we only found FDR-corrected significant results at the network level, we also observed strong correlations at the ROI level. However, the lack of a specific hypothesis for selecting brain regions in the ROI-level analysis may have impacted its efficacy, while the network-based approach was more contingent as it helped to limit our search space and identify significant results.

Nevertheless, the absence of significant correlations between EFs and behavioral outcomes highlights the need for further investigation to fully understand how EF-induced neural changes translate into subjective experiences and behaviors. Future research should delve deeper into the underlying mechanisms of these relationships, explore additional analytical approaches, and consider other factors that may influence the effects of EFs on brain function. By addressing these avenues, we can advance our knowledge and maximize the potential of EF-based interventions for both clinical and cognitive applications.

## Supporting information

S1 Text Inclusion/Exclusion criteria. (DOCX)

S2 Text Imaging parameters. (DOCX)

S3 Text General information about EF distribution patterns. (DOCX)

S4 Text Craving scores. Changes in behavioral scores in terms of craving score were quantified based on VAS scores immediately before and after each MRI session in both active (right side) and sham (left side) groups.
(DOCX)

## References

[1] Mosayebi-Samani M, Jamil A, Salvador R, Ruffini G, Haueisen J, Nitsche MA. The impact of individual electrical fields and anatomical factors on the neurophysiological outcomes of tDCS: A TMS-MEP and MRI study. Brain Stimulation. 2021;14(2):316–26. doi: 10.1016/j.brs.2021.01.016 33516860

[2] Kim J-H, Kim D-W, Chang WH, Kim Y-H, Kim K, Im C-H. Inconsistent outcomes of transcranial direct current stimulation may originate from anatomical differences among individuals: electric field simulation using individual MRI data. Neuroscience letters. 2014;564:6–10. doi: 10.1016/j.neulet.2014.01.054 24508704

[3] Nitsche MA, Paulus W. Excitability changes induced in the human motor cortex by weak transcranial direct current stimulation. The Journal of physiology. 2000;527(Pt 3):633. doi: 10.1111/j.1469-7793.2000.t01-1-00633.x 10990547 PMC2270099

[4] Regner GG, Pereira P, Leffa DT, De Oliveira C, Vercelino R, Fregni F, et al. Preclinical to clinical translation of studies of transcranial direct-current stimulation in the treatment of epilepsy: a systematic review. Frontiers in neuroscience. 2018;12:189. doi: 10.3389/fnins.2018.00189 29623027 PMC5874505

[5] Palm U, Hasan A, Strube W, Padberg F. tDCS for the treatment of depression: a comprehensive review. European archives of psychiatry and clinical neuroscience. 2016;266:681–94. doi: 10.1007/s00406-016-0674-9 26842422

[6] Lauro LJR, Rosanova M, Mattavelli G, Convento S, Pisoni A, Opitz A, et al. TDCS increases cortical excitability: Direct evidence from TMS–EEG. Cortex. 2014;58:99–111. doi: 10.1016/j.cortex.2014.05.003 24998337

[7] Li LM, Uehara K, Hanakawa T. The contribution of interindividual factors to variability of response in transcranial direct current stimulation studies. Frontiers in cellular neuroscience. 2015;9:181. doi: 10.3389/fncel.2015.00181 26029052 PMC4428123

[8] Hill AT, Fitzgerald PB, Hoy KE. Effects of anodal transcranial direct current stimulation on working memory: a systematic review and meta-analysis of findings from healthy and neuropsychiatric populations. Brain stimulation. 2016;9(2):197–208. doi: 10.1016/j.brs.2015.10.006 26597929

[9] Horvath JC, Forte JD, Carter O. Quantitative review finds no evidence of cognitive effects in healthy populations from single-session transcranial direct current stimulation (tDCS). Brain stimulation. 2015;8(3):535–50. doi: 10.1016/j.brs.2015.01.400 25701175

[10] Wiethoff S, Hamada M, Rothwell JC. Variability in response to transcranial direct current stimulation of the motor cortex. Brain stimulation. 2014;7(3):468–75. doi: 10.1016/j.brs.2014.02.003 24630848

[11] Laakso I, Mikkonen M, Koyama S, Hirata A, Tanaka S. Can electric fields explain inter-individual variability in transcranial direct current stimulation of the motor cortex? Scientific reports. 2019;9(1):626. doi: 10.1038/s41598-018-37226-x 30679770 PMC6345748

[12] Laakso I, Tanaka S, Koyama S, De Santis V, Hirata A. Inter-subject variability in electric fields of motor cortical tDCS. Brain stimulation. 2015;8(5):906–13. doi: 10.1016/j.brs.2015.05.002 26026283

[13] Gomez-Tames J, Asai A, Mikkonen M, Laakso I, Tanaka S, Uehara S, et al. Group-level and functional-region analysis of electric-field shape during cerebellar transcranial direct current stimulation with different electrode montages. Journal of neural engineering. 2019;16(3):036001. doi: 10.1088/1741-2552/ab0ac5 30808008

[14] Datta A, Bansal V, Diaz J, Patel J, Reato D, Bikson M. Gyri-precise head model of transcranial direct current stimulation: improved spatial focality using a ring electrode versus conventional rectangular pad. Brain stimulation. 2009;2(4):201–7. e1. doi: 10.1016/j.brs.2009.03.005 20648973 PMC2790295

[15] Huang Y, Liu AA, Lafon B, Friedman D, Dayan M, Wang X, et al. Measurements and models of electric fields in the in vivo human brain during transcranial electric stimulation. elife. 2017;6:e18834. doi: 10.7554/eLife.18834 28169833 PMC5370189

[16] Opitz A, Legon W, Rowlands A, Bickel WK, Paulus W, Tyler WJ. Physiological observations validate finite element models for estimating subject-specific electric field distributions induced by transcranial magnetic stimulation of the human motor cortex. Neuroimage. 2013;81:253–64. doi: 10.1016/j.neuroimage.2013.04.067 23644000

[17] Edwards D, Cortes M, Datta A, Minhas P, Wassermann EM, Bikson M. Physiological and modeling evidence for focal transcranial electrical brain stimulation in humans: a basis for high-definition tDCS. Neuroimage. 2013;74:266–75. doi: 10.1016/j.neuroimage.2013.01.042 23370061 PMC4359173

[18] Jog MV, Smith RX, Jann K, Dunn W, Lafon B, Truong D, et al. In-vivo imaging of magnetic fields induced by transcranial direct current stimulation (tDCS) in human brain using MRI. Scientific reports. 2016;6(1):34385. doi: 10.1038/srep34385 27698358 PMC5048181

[19] Kasten FH, Duecker K, Maack MC, Meiser A, Herrmann CS. Integrating electric field modelling and neuroimaging to explain inter-individual variability of tACS effects. BioRxiv. 2019:581207. 10.1038/s41467-019-13417-6 PMC6882891 31780668

[20] Saturnino GB, Puonti O, Nielsen JD, Antonenko D, Madsen KH, Thielscher A. SimNIBS 2.1: a comprehensive pipeline for individualized electric field modelling for transcranial brain stimulation. Brain and human body modeling: computational human modeling at EMBC 2018. 2019:3–25. 31725247

[21] Huang Y, Datta A, Bikson M, Parra LC, editors. ROAST: an open-source, fully-automated, realistic volumetric-approach-based simulator for TES. 2018 40th Annual International Conference of the IEEE Engineering in Medicine and Biology Society (EMBC); 2018: IEEE. 10.1109/EMBC.2018.8513086 30441043

[22] Lang S, Gan LS, McLennan C, Kirton A, Monchi O, Kelly JJ. Preoperative transcranial direct current stimulation in glioma patients: a proof of concept pilot study. Frontiers in neurology. 2020;11:593950. doi: 10.3389/fneur.2020.593950 33329346 PMC7710969

[23] Kar K, Ito T, Cole MW, Krekelberg B. Transcranial alternating current stimulation attenuates BOLD adaptation and increases functional connectivity. Journal of neurophysiology. 2020. doi: 10.1152/jn.00376.2019 31825706 PMC6985864

[24] Antonenko D, Thielscher A, Saturnino GB, Aydin S, Ittermann B, Grittner U, et al. Towards precise brain stimulation: Is electric field simulation related to neuromodulation? Brain stimulation. 2019;12(5):1159–68. doi: 10.1016/j.brs.2019.03.072 30930209

[25] Esmaeilpour Z, Shereen AD, Ghobadi-Azbari P, Datta A, Woods AJ, Ironside M, et al. Methodology for tDCS integration with fMRI. Human brain mapping. 2020;41(7):1950–67. doi: 10.1002/hbm.24908 31872943 PMC7267907

[26] Indahlastari A, Albizu A, Kraft JN, O’Shea A, Nissim NR, Dunn AL, et al. Individualized tDCS modeling predicts functional connectivity changes within the working memory network in older adults. Brain stimulation. 2021;14(5):1205–15. doi: 10.1016/j.brs.2021.08.003 34371212 PMC8892686

[27] Jamil A, Batsikadze G, Kuo HI, Meesen RL, Dechent P, Paulus W, et al. Current intensity-and polarity-specific online and aftereffects of transcranial direct current stimulation: An fMRI study. Human brain mapping. 2020;41(6):1644–66. doi: 10.1002/hbm.24901 31860160 PMC7267945

[28] Abellaneda-Pérez K, Vaqué-Alcázar L, Perellón-Alfonso R, Solé-Padullés C, Bargalló N, Salvador R, et al. Multifocal transcranial direct current stimulation modulates resting-state functional connectivity in older adults depending on the induced current density. Frontiers in aging neuroscience. 2021;13:725013. doi: 10.3389/fnagi.2021.725013 34899266 PMC8662695

[29] Preisig BC, Hervais-Adelman A. The predictive value of individual electric field modeling for transcranial alternating current stimulation induced brain modulation. Frontiers in Cellular Neuroscience. 2022;16:818703. doi: 10.3389/fncel.2022.818703 35273479 PMC8901488

[30] Halko M, Datta A, Plow EB, Scaturro J, Bikson M, Merabet LB. Neuroplastic changes following rehabilitative training correlate with regional electrical field induced with tDCS. Neuroimage. 2011;57(3):885–91. doi: 10.1016/j.neuroimage.2011.05.026 21620985 PMC3167218

[31] Yuan K, Ti C-hE, Wang X, Chen C, Lau CC-y, Chu WC-w, et al. Individual electric field predicts functional connectivity changes after anodal transcranial direct-current stimulation in chronic stroke. Neuroscience Research. 2023;186:21–32. doi: 10.1016/j.neures.2022.10.003 36220454

[32] Steinmann I, Williams KA, Wilke M, Antal A. Detection of Transcranial Alternating Current Stimulation Aftereffects Is Improved by Considering the Individual Electric Field Strength and Self-Rated Sleepiness. Frontiers in Neuroscience. 2022;16:870758. doi: 10.3389/fnins.2022.870758 35833087 PMC9272587

[33] Ekhtiari H, Soleimani G, Kuplicki R, Yeh HW, Cha YH, Paulus M. Transcranial direct current stimulation to modulate fMRI drug cue reactivity in methamphetamine users: a randomized clinical trial. Human Brain Mapping. 2022;43(17):5340–57. doi: 10.1002/hbm.26007 35915567 PMC9812244

[34] Rossini PM, Burke D, Chen R, Cohen L, Daskalakis Z, Di Iorio R, et al. Non-invasive electrical and magnetic stimulation of the brain, spinal cord, roots and peripheral nerves: basic principles and procedures for routine clinical and research application. An updated report from an IFCN Committee. Clinical neurophysiology. 2015;126(6):1071–107. 25797650 10.1016/j.clinph.2015.02.001 PMC6350257

[35] Opitz A, Paulus W, Will S, Antunes A, Thielscher A. Determinants of the electric field during transcranial direct current stimulation. Neuroimage. 2015;109:140–50. doi: 10.1016/j.neuroimage.2015.01.033 25613437

[36] Geuzaine C, Remacle JF. Gmsh: A 3-D finite element mesh generator with built-in pre-and post-processing facilities. International journal for numerical methods in engineering. 2009;79(11):1309–31.

[37] Huang Y, Datta A, Bikson M, Parra LC. Realistic volumetric-approach to simulate transcranial electric stimulation—ROAST—a fully automated open-source pipeline. Journal of neural engineering. 2019;16(5):056006. doi: 10.1088/1741-2552/ab208d 31071686 PMC7328433

[38] Puonti O, Saturnino GB, Madsen KH, Thielscher A. Value and limitations of intracranial recordings for validating electric field modeling for transcranial brain stimulation. Neuroimage. 2020;208:116431. doi: 10.1016/j.neuroimage.2019.116431 31816421

[39] Kwon OI, Sajib SZ, Sersa I, Oh TI, Jeong WC, Kim HJ, et al. Current density imaging during transcranial direct current stimulation using DT-MRI and MREIT: algorithm development and numerical simulations. IEEE Transactions on Biomedical Engineering. 2015;63(1):168–75. doi: 10.1109/TBME.2015.2448555 26111387

[40] Jog M, Jann K, Yan L, Huang Y, Parra L, Narr K, et al. Concurrent imaging of markers of current flow and neurophysiological changes during tDCS. Frontiers in Neuroscience. 2020;14:374. doi: 10.3389/fnins.2020.00374 32372913 PMC7186453

[41] Joy ML, editor MR current density and conductivity imaging: the state of the Aart. The 26th annual international conference of the IEEE engineering in medicine and biology society; 2004: IEEE. 10.1109/IEMBS.2004.1404484 17271541

[42] Fan L, Li H, Zhuo J, Zhang Y, Wang J, Chen L, et al. The human brainnetome atlas: a new brain atlas based on connectional architecture. Cerebral cortex. 2016;26(8):3508–26. doi: 10.1093/cercor/bhw157 27230218 PMC4961028

[43] Bates D. Fitting linear mixed models in R. R news. 2005;5(1):27–30.

[44] Whitfield-Gabrieli S, Nieto-Castanon A. Conn: a functional connectivity toolbox for correlated and anticorrelated brain networks. Brain connectivity. 2012;2(3):125–41. doi: 10.1089/brain.2012.0073 22642651

[45] Heller R, Stanley D, Yekutieli D, Rubin N, Benjamini Y. Cluster-based analysis of FMRI data. NeuroImage. 2006;33(2):599–608. doi: 10.1016/j.neuroimage.2006.04.233 16952467

[46] Esmaeilpour Z, Shereen AD, Ghobadi-Azbari P, Datta A, Woods AJ, Ironside M, et al. Methodology for tDCS integration with fMRI. Human Brain Mapping. 2019. doi: 10.1002/hbm.24908 31872943 PMC7267907

[47] Soleimani G, Towhidkhah F, Saviz M, Ekhtiari H. Cortical morphology in cannabis use disorder: implications for transcranial direct current stimulation treatment. Basic and Clinical Neuroscience. 2021:0-. 10.32598/bcn.2021.3400.1 PMC11016884 38628838

[48] McCalley DM, Hanlon CA. Regionally specific gray matter volume is lower in alcohol use disorder: Implications for noninvasive brain stimulation treatment. Alcoholism: clinical and experimental research. 2021;45(8):1672–83. doi: 10.1111/acer.14654 34120347 PMC8560006

[49] Mizutani-Tiebel Y, Takahashi S, Karali T, Mezger E, Bulubas L, Papazova I, et al. Differences in electric field strength between clinical and non-clinical populations induced by prefrontal tDCS: A cross-diagnostic, individual MRI-based modeling study. NeuroImage: Clinical. 2022;34:103011. doi: 10.1016/j.nicl.2022.103011 35487132 PMC9125784

[50] Berker AOd, Bikson M, Bestmann S. Predicting the behavioral impact of transcranial direct current stimulation: issues and limitations. Frontiers in human neuroscience. 2013;7:613. doi: 10.3389/fnhum.2013.00613 24109445 PMC3790257

[51] Jiang J, Truong DQ, Esmaeilpour Z, Huang Y, Badran BW, Bikson M. Enhanced tES and tDCS computational models by meninges emulation. Journal of neural engineering. 2020;17(1):016027. doi: 10.1088/1741-2552/ab549d 31689695 PMC7254922

[52] Schmidt C, Wagner S, Burger M, van Rienen U, Wolters CH. Impact of uncertain head tissue conductivity in the optimization of transcranial direct current stimulation for an auditory target. Journal of neural engineering. 2015;12(4):046028. doi: 10.1088/1741-2560/12/4/046028 26170066 PMC4539365

[53] Saturnino GB, Thielscher A, Madsen KH, Knösche TR, Weise K. A principled approach to conductivity uncertainty analysis in electric field calculations. Neuroimage. 2019;188:821–34. doi: 10.1016/j.neuroimage.2018.12.053 30594684

[54] Ye H, Steiger A. Neuron matters: electric activation of neuronal tissue is dependent on the interaction between the neuron and the electric field. Journal of neuroengineering and rehabilitation. 2015;12:1–9. 26265444 10.1186/s12984-015-0061-1 PMC4534030

[55] Huang WA, Stitt IM, Negahbani E, Passey D, Ahn S, Davey M, et al. Transcranial alternating current stimulation entrains alpha oscillations by preferential phase synchronization of fast-spiking cortical neurons to stimulation waveform. Nature communications. 2021;12(1):3151. doi: 10.1038/s41467-021-23021-2 34035240 PMC8149416

[56] Zhang B, Yuan X, Lv H, Che J, Wang S, Shang P. Biophysical mechanisms underlying the effects of static magnetic fields on biological systems. Progress in Biophysics and Molecular Biology. 2022. doi: 10.1016/j.pbiomolbio.2022.09.002 36240898

[57] Ekhtiari H, Ghobadi-Azbari P, Thielscher A, Antal A, Li LM, Shereen AD, et al. A checklist for assessing the methodological quality of concurrent tES-fMRI studies (ContES checklist): a consensus study and statement. Nature protocols. 2022;17(3):596–617. doi: 10.1038/s41596-021-00664-5 35121855 PMC7612687

[58] Rahman A, Toshev PK, Bikson M. Polarizing cerebellar neurons with transcranial direct current stimulation. 2014. p. 435–8. 10.1016/j.clinph.2013.10.003 24176296

[59] Bikson M, Inoue M, Akiyama H, Deans JK, Fox JE, Miyakawa H, et al. Effects of uniform extracellular DC electric fields on excitability in rat hippocampal slices in vitro. The Journal of physiology. 2004;557(1):175–90. doi: 10.1113/jphysiol.2003.055772 14978199 PMC1665051

[60] Deans JK, Powell AD, Jefferys JG. Sensitivity of coherent oscillations in rat hippocampus to AC electric fields. The Journal of physiology. 2007;583(2):555–65. doi: 10.1113/jphysiol.2007.137711 17599962 PMC2277040

[61] Mulyana B, Tsuchiyagaito A, Misaki M, Kuplicki R, Smith J, Soleimani G, et al. Online closed-loop real-time tES-fMRI for brain modulation: A technical report. Brain and Behavior. 2022;12(10):e2667. doi: 10.1002/brb3.2667 36134450 PMC9575607

[62] Soleimani G, Nitsche MA, Bergmann TO, Towhidkhah F, Violante I, Lorenz R, et al. Closing the loop between brain and electrical stimulation: Towards precision neuromodulation treatments. 2022. 10.1038/s41398-023-02565-5 PMC10427701 37582922

