# Supplementary material for: Dose-response in modulating brain function with transcranial direct current stimulation: From local to network levels
Source: PLoS Comput Biol. 2023 Oct 26;19(10):e1011572. doi: 10.1371/journal.pcbi.1011572 (PMC10629666; doi:10.1371/journal.pcbi.1011572)
Supplement: S1 Text — (DOCX) [file pcbi.1011572.s001.docx]

**S.1. Inclusion/Exclusion criteria**

The inclusion criteria for this study were as follows: (1) proficiency in English, (2) diagnosed with methamphetamine use disorder within the past 12 months, (3) enrolled in a residential abstinence-based treatment program for methamphetamine use disorder, (4) abstinent from methamphetamine for at least one week, and (5) willing and capable of participating in the informed consent process. Exclusion criteria encompassed: (1) unwillingness or inability to complete essential study components, including magnetic resonance imaging (e.g., due to claustrophobia), drug cue rating, or behavioral assessment, (2) reported abstinence from methamphetamine for more than 6 months, based on self-report, (3) diagnosis of schizophrenia or bipolar disorder according to the MINI interview, (4) presence of active suicidal ideation with intent or plan, as determined by self-report or assessment by the principal investigator or study staff during the initial screening or any subsequent phase of the study, and (5) positive drug test results for amphetamines, opioids, cannabis, alcohol, phencyclidine, or cocaine, confirmed by breath analyzer and urine tests.
