# Supplementary material for: Dose-response in modulating brain function with transcranial direct current stimulation: From local to network levels
Source: PLoS Comput Biol. 2023 Oct 26;19(10):e1011572. doi: 10.1371/journal.pcbi.1011572 (PMC10629666; doi:10.1371/journal.pcbi.1011572)
Supplement: S2 Text — (DOCX) [file pcbi.1011572.s002.docx]

**S.2. Imaging parameters**

Structural and functional MRIs were obtained on two identical GE MRI 750 3T scanners. Structural MRI parameters: TR/TE = 5/2.012 ms, FOV/slice = 24 x 192/0.9 mm, 256x256 matrix producing 0.938 x 0.9 mm voxels and 186 axial slices for T1-weighted images and TR/TE=8108/137.728ms, FOV/slice=240/2mm, 512x512 matrix producing 0.469x0.469x2mm voxels and 80 coronal slices for T2-weighted images. T1- and T2-weighted MR images were used for generating computational head models for each individual. Task-state fMRI parameters: TR/TE = 2000/27 ms, FOV/slice = 240/2.9 mm, 128x128 matrix producing 1.857x1.857x2.9 mm voxels, 39 axial slices, and 196 repetitions.
