# Supplementary material for: Dose-response in modulating brain function with transcranial direct current stimulation: From local to network levels
Source: PLoS Comput Biol. 2023 Oct 26;19(10):e1011572. doi: 10.1371/journal.pcbi.1011572 (PMC10629666; doi:10.1371/journal.pcbi.1011572)
Supplement: S3 Text — (DOCX) [file pcbi.1011572.s003.docx]

**S.3. General information about EF distribution patterns**

The EF hotspot, representing the spatial global maximum, was determined by calculating the 99th percentile across the entire brain for each participant. A notable variation in maximum EFs was observed among individuals using the F4-Fp1 electrode arrangement targeting the dorsolateral prefrontal cortex (DLPFC), with values ranging from 0.28 to 0.58 V/m (mean ± SD: 0.41 ± 0.07 V/m). Individual and group-level analysis of personalized head models revealed that the EF peaks (99th percentile) were not situated directly beneath the stimulating electrode (F4), but rather within the frontopolar area. The frontopolar area, primarily corresponding to Brodmann's area 10, occupies the anterior portion of the frontal lobe, while the DLPFC, encompassing Brodmann's areas 9 and 46, is located on the lateral and dorsal part of the medial convexity of the frontal lobe. The mean location of the EF peaks, expressed in MNI space, was [-2.62, 49.00, -4.54], with a standard deviation of [7.05, 7.71, 8.94]. At the group level, the average EF strength was 0.38 ± 0.04 V/m, ranging from 0.28 to 0.59 V/m.
