# Supplementary material for: Dose-response in modulating brain function with transcranial direct current stimulation: From local to network levels
Source: PLoS Comput Biol. 2023 Oct 26;19(10):e1011572. doi: 10.1371/journal.pcbi.1011572 (PMC10629666; doi:10.1371/journal.pcbi.1011572)
Supplement: S4 Text — Changes in behavioral scores in terms of craving score were quantified based on VAS scores immediately before and after each MRI session in both active (right side) and sham (left side) groups. (DOCX) [file pcbi.1011572.s004.docx]

**S.4. Behavioral Results**

**Fig A. Craving scores.** Changes in behavioral scores in terms of craving score were quantified based on VAS scores immediately before and after each MRI session in both active (right side) and sham (left side) groups.
